# Optimizing Maternal Nutrition: The Importance of a Tailored Approach

**DOI:** 10.1093/cdn/nzac118

**Published:** 2022-07-22

**Authors:** Lauren R Brink, Tonya M Bender, Rosalind Davies, Hanqi Luo, Derek Miketinas, Neil Shah, Nik Loveridge, Gabriele Gross, Neil Fawkes

**Affiliations:** Medical and Scientific Affairs, Reckitt/Mead Johnson Nutrition Institute, Evansville, IN, USA; Medical and Scientific Affairs, Reckitt/Mead Johnson Nutrition Institute, Evansville, IN, USA; Medical and Scientific Affairs, Reckitt/Mead Johnson Nutrition Institute, Slough, UK; Emory University, Atlanta, GA, USA; Nutrition and Food Sciences, Texas Woman's University, Houston, TX, USA; Medical and Scientific Affairs, Reckitt/Mead Johnson Nutrition Institute, Slough, UK; Medical and Scientific Affairs, Reckitt/Mead Johnson Nutrition Institute, Slough, UK; Medical and Scientific Affairs, Reckitt/Mead Johnson Nutrition Institute, Nijmegen, The Netherlands; Medical and Scientific Affairs, Reckitt/Mead Johnson Nutrition Institute, Slough, UK

**Keywords:** pregnancy, nutrition, personalization, tailored approach, supplements

## Abstract

Improving nutritional status during pregnancy is a global interest. Frequently, women either fail to meet or exceed nutrient recommendations. Current strategies to improve maternal nutrition focus on a “one-size-fits-all” approach and fail to consider individual factors that affect the mother's overall nutritional status. The objectives of this review were to determine the importance of key nutrients for optimal maternal and fetal health, to explore to what extent current recommendations consider individual factors, and to explore novel strategies to close the gap between current guidelines and real-world challenges through more personalized approaches. This review intercalated different nutritional guidelines and recent scientific publications and research initiatives related to maternal nutrition. Based on that, an overview of current recommendations, challenges related to present approaches, and perspectives for future directions are described. Current guidelines are not optimally supporting adequate nutrient intake and health of expectant mothers and their offspring. Existing recommendations are not consistent and do not sufficiently take into account how interindividual variation leads to differences in nutrient status. Personalized nutrition offers women the opportunity to improve their health by using strategies that are tailored to their unique nutritional needs. Such strategies can include personalized supplementation, holistic lifestyle interventions, digital and application-based technologies, and dietary assessment through blood biomarker and genetic analysis. However, these approaches warrant further investigation and optimization. More personalized approaches have the potential to optimize mothers’ and their offspring's health outcomes more appropriately to their nutritional needs before, during, and after pregnancy. Moving away from a generalized “one-size-fits-all” approach can be achieved through a variety of means. Future aims should be to provide supporting evidence to create customized subpopulation-based or individualized recommendations, improve nutrition education, and develop novel approaches to improve adherence to dietary and lifestyle interventions.

## Teaser

Personalized nutrition has the potential to improve mothers’ and offspring's health outcomes by using strategies that are appropriate for the mother's individual nutritional needs before, during, and after pregnancy.

## Introduction

The developmental processes that span pregnancy and lactation are associated with significant maternal changes in metabolism and physiology, both of which support the developing fetus and help to prepare the mother for childbirth and breastfeeding (1, 2). The gestational experience is substantially influenced by a woman's age and overall state of health. Adequate nutrition during conception, pregnancy, and lactation is vital for good maternal health, as well as to support fetal growth and development throughout pregnancy and beyond (3–5). In the shorter term, imbalances in maternal intake of critical nutrients can result in adverse pregnancy outcomes, including low birth weight, poor fetal growth, and fetal malformations such as neural tube and congenital heart defects (6, 7), as well as exacerbate existing micronutrient deficiencies. Additionally, poor maternal nutritional status can substantially increase the risk of developing chronic health conditions in offspring later in life, such as obesity, diabetes, heart disease, and noncommunicable diseases (8, 9). Considering the potentially life-long implications of in utero conditions on offspring health (10), it is necessary to understand and meet the health demands of women during all stages of pregnancy while ensuring that any guidance issued to them is scientifically validated.

The nutritional demands of expectant mothers differ notably compared with women who are not pregnant. Existing dietary guidelines aim to establish healthy eating and lifestyle practices in the general population; however, these demands are often not met (11, 12). Despite the importance of adequate nutrient status, women face different barriers to achieving optimal nutrition due to differences in socioeconomic status, diet quality, food availability, and frequency of reproduction (13). Globally, malnutrition poses as a double burden, in which both insufficient and excess nutrient intakes coexist; consequently, obesity and malnutrition are no longer associated with solely high- and low-income countries, respectively (8). However, limited data on nutrient intake from pregnant women in low- and middle-income countries exist (14). Therefore, many interventions and strategies to potentially improve maternal nutrition may only have widespread feasibility in high-income settings.

Given the numerous complex and often interplaying factors that affect nutrition, recent evidence supports the use of personalized strategies that consider the unique needs of the mother to improve maternal and fetal outcomes. In this review, current nutrient recommendations in pregnancy are assessed along with the challenges with closing the existing gaps between guidelines and real-world practices. Factors affecting the complexity of achieving optimal nutritional status are presented, and strategies to address individualized needs to achieve optimal maternal and fetal health are proposed. Due to the higher prevalence of data from high-income countries, these are the primary focus of the review, with discussion pertaining to the challenges when applying proposed personalized approaches in low- and middle-income populations.

## Nutrient Requirements in Pregnancy

There is an increasing body of evidence that highlights the link between adequate nutrition during the first thousand days of life and the risk of future chronic disease for both the mother and infant (15). As such, recommendations for micronutrients and macronutrients along with the risks of excess nutrient intake (16) must all be considered to ensure adequate support of the developing fetus, as well as preparing the mother for childbirth and lactation. The roles of key nutrients during pregnancy along with their respective risks relating to deficiency are presented in **Table 1**, whereas a summary of key micro- and macronutrients and DRIs for nonpregnant, pregnant, and lactating women across 3 age ranges (14–18 y, 19–30 y, and 31–50 y), as recommended by the National Academy of Sciences, Engineering, and Medicine (NAS) are presented in **Table 2**.

**TABLE 1:** Roles and biological effects of inadequacy of nutrients during pregnancy

Nutrient	Role^1^	Biological effects of inadequacy
Micronutrients (vitamins and minerals)		
Calcium	Critical for bone/skeletal development, supports muscle function, nerve impulse transmission, and hormone secretion	Potentially low fetal bone mineralization, osteopenia and pre-eclampsia/hypertension in pregnancy, muscle cramps
Folate	Supports neural tube formation and cognitive function, involved in protein and DNA synthesis, supports erythropoiesis	Neural tube defects, anemia in pregnancy, congenital malformations low birth weight
Iodine	Supports fetal and maternal thyroid function, fetal brain development; regulates growth, development, and metabolism	Neurodevelopmental delay/mental impairment
Iron	Critical for hemoglobin synthesis and cellular and organ function	Abnormal cognitive development and function, low birth weight/small for gestational age, birth defects, preterm delivery, anemia in pregnancy
Vitamin A	Supports visual development, growth, immunity, and organ development	Visual impairment, birth defects, intrauterine growth restriction, maternal night blindness
Vitamin B-6	Regulates DNA methylation, energy generation, and blood cell formation, supports enzyme function	Anemia in pregnancy, birth defects
Vitamin B-12	Plays a role in methylation of DNA, proteins, and neurotransmitters, supports erythropoiesis and brain development	Birth defects, low birth weight, neuropsychiatric problems
Vitamin C	Enhances iron uptake, supports bone and teeth health, serves as an antioxidant, and supports collagen synthesis	Negative fetal brain effects
Vitamin D	Critical for bone growth, supports immune and nervous system function, gene expression, and glucose metabolism	Rickets and bone fractures, risk of small for gestational age, gestational diabetes mellitus
Vitamin E	Protects against oxidative stress	Poor fetal and maternal outcomes
Vitamin K	Aids in blood clotting	Hemorrhaging/excessive bleeding
Thiamin	Supports muscle function and nervous system, blood cell formation	Impaired fetal brain development
Riboflavin	Energy generation, blood cell formation	Pre-eclampsia, risk of congenital heart defects
Macronutrients		
Carbohydrate	Supports fetal growth, promotes healthy digestion, organ and muscle function	Restricted fetal growth
Fat^2^	Fetal neurological development, cell signaling, growth	Inadequate fetal brain and eye development
Protein	Building blocks for cell components	Restricted fetal growth
Total water	Supports amniotic fluid and blood circulation, helps with increased maternal blood volume	Low amniotic fluid, potential birth defects

1 Sources: Hanson et al., 2015 (53); Kominiarek and Rajan, 2016 (65); Mousa et al., 2019 (118).

2 Including ɷ-3 fatty acids.

**TABLE 2:** NAS daily requirements of nutrients of concern in non-pregnant, pregnant, and lactating women across three age groups

	Dietary Reference Intakes (Daily): Recommended Dietary Allowances and Adequate Intakes by age range (years)									
	Non-pregnant				Pregnancy			Lactation		
Nutrient		14–18	19–30	31–50	14–18	19–30	31–50	14–18	19–30	31–50
Micronutrients (Vitamins and Minerals)	Calcium (mg)	1,300	1,000	1,000	1,300*	1,000*	1,000*	1,300*	1,000*	1,000*
	Folate^a^ (µg)	400	400	400	600**	600**	600**	500**	500**	500**
	Iodine (µg)	150	150	150	220**	220**	220**	290**	290**	290**
	Iron (mg)	15	18	18	27**	27**	27**	10***	9***	9***
	Vitamin A (µg)	700	700	700	750**	770**	770**	1,200**	1,300**	1,300**
	Vitamin B6 (mg)	1.2	1.3	1.3	1.9**	1.9**	1.9**	2.0**	2.0**	2.0**
	Vitamin B12(µg)	2.4	2.4	2.4	2.6**	2.6**	2.6**	2.8**	2.8**	2.8**
	Vitamin C (mg)	65	75	75	80**	85**	85**	115**	120**	120**
	Vitamin D(µg)	15	15	15	15*	15*	15*	15*	15*	15*
	Vitamin E^b^ (mg)	15	15	15	15*	15*	15*	19**	19**	19**
	Vitamin K^c^ (µg)	75	90	90	75*	90*	90*	75*	90*	90*
	Thiamin (mg)	1.0	1.1	1.1	1.4**	1.4**	1.4**	1.4**	1.4**	1.4**
	Riboflavin (mg)	1.0	1.1	1.1	1.4**	1.4**	1.4**	1.6**	1.6**	1.6**
Macronutrients	Carbohydrate (g)	130	130	130	175**	175**	175**	210**	210**	210**
	Omega-3 fatty acids^d^ (g)	1.1	1.1	1.1	1.4**	1.4**	1.4**	1.3**	1.3**	1.3**
	Protein (g)	46	46	46	71**	71**	71**	71**	71**	71**
	Total water (L)	2.3	2.7	2.7	3.0**	3.0**	3.0**	3.8**	3.8**	3.8**

NAS = National Academy of Sciences, Engineering, and Medicine

With the exception of Total Water & Omega-3 fatty acids (Adequate Intakes (AI), these values represent the Recommended Dietary Allowances (RDAs).

a Values reported as dietary folate equivalents. 1 dietary equivalent = 1 µg food folate = 0.6 µg of folic acid from fortified food or as a supplement consumed with food = 0.5 µg if a supplement taken on an empty stomach.

b Values presented are for vitamin E as α-tocopherol.

c Values obtained from: Institute of Medicine (US) Panel on Micronutrients. Washington (DC): National Academies Press (US); 2001.

d Values presented for alpha-linolenic acid, the most common dietary omega-3 fatty acid.

Note: Astericks are provided for pregnancy and lactation values are color-coded to designate if values are the same (*), higher (**) or lower (***) than non-pregnant values.

Note: Unless otherwise specified, values presented are taken from documents issued by the Food and Nutrition Board of the Institute of Medicine, National Academy of Sciences available at: https://ods.od.nih.gov/HealthInformation/Dietary_Reference_Intakes.aspx.

Strategies to improve maternal nutrition should avoid the “more is better” approach. Overconsumption of certain nutrients can pose significant problems, especially in individuals who are already achieving sufficient intake. Vitamin A is an example of a micronutrient that plays an important role during fetal development; however, its intake should be monitored closely during pregnancy to ensure that intake does not exceed upper limits. During pregnancy, vitamin A is essential for embryonic ocular and bone development as well as supporting the immune system functions (17, 18). Excessive dietary consumption or overuse of vitamin A–containing retinol creams could be teratogenic, particularly during the first trimester, thereby increasing the risk of inducing severe fetal developmental abnormalities (17, 19); B-carotene, a precursor of vitamin A, has long been considered a much less toxic and safer source of vitamin A (20). A recent study investigating the usual dietary intake of pregnant women in the United States found that women were exceeding the Reference Daily Intakes of several nutrients, notably iron and folate, once supplement use had been included in nutrient estimates (11). Excess intake of iron midpregnancy may restrict fetal growth (21), whereas high folate may exacerbate neurological damage in vitamin B-12–deficient individuals (22). It is important for women and health care professionals (HCPs)/Registered Dietitian Nutritionists (RDNs) to avoid a “more is better” approach to minimize any potential adverse outcomes while more extensive research is undertaken in pregnant populations.

Current understanding of nutrient requirements in pregnant and lactating women is limited and thus recommendations may not be accurate in all cases. A recent review of studies assessing nutrient reference values highlighted how pregnant and lactating women are severely underrepresented in research efforts and were included in only 17% of 704 studies analyzed (23). The authors also stressed that, while nutrient reference values are intended to guide the general population, the research underpinning these values may not be generalizable to many subpopulations; for instance, information on race and ethnicity was not recorded in over 90% of the studies (23). Future high-quality studies with robust trial designs and research methods, including women from more diverse populations, are warranted in order to advance and disseminate improved knowledge in this area (23, 24).

## Challenges Related to Current Guidelines for Maternal Nutrition

Nutritional guidance from leading scientific organizations and government agencies covers the general, daily requirements for most nutrients, including recommendations relating to specific life stages, such as pregnancy and lactation. Guidance generally remains broad, with little focus on subpopulation requirements, supplementation use, or individual needs. Furthermore, multiple public health agencies often provide different recommendations that are applicable to the same population, which can often be confusing or contradictory. For instance, there are differences in guidelines from the WHO, NAS (previously known as Institute of Medicine), and American College of Obstetricians and Gynecologists (ACOG) recommendations, all of which apply to women in the United States (**Table 3**). Daily calcium supplement recommendations, for example, are notably lower for the NAS (250 mg) compared with WHO (1500–2000 mg) and ACOG (≤1300 mg). The NAS recommends supplementation with 7 key nutrients, with additional recommendations under specific conditions (Table 3). Finally, the ACOG recommends folate and calcium supplementation and 2 servings of fish per week for all pregnant women (Table 3). Such inconsistencies and contradictions can be very confusing to HCPs/RDNs as well as consumers and leave women unsure as to how they should optimize their nutrition during pregnancy, whether through diet, supplements, or otherwise.

**TABLE 3:** Expert recommendations for daily supplementation during pregnancy^1^

Nutrient	WHO^2^	NAS^3^	ACOG^4^
Vitamin A	Only in populations where deficiency is a severe public health problem	—	—
Vitamin B-6	Recommended for nausea (T1)	2 mg	—
Vitamin C	Not recommended	50 mg	—
Vitamin D	Not recommended	5 µg	–
Folic acid, DFE	400 µg	300 µg DFE	600 µg from all sources, supplementation recommended
Calcium	1500–2000 mg in populations with low calcium intake	250 mg	1000 mg, 1300 mg
Iron	30–60 mg	30 mg	—
Zinc	Context specific	15 mg	—
DHA	—	—	2 servings of fish per week
Copper	—	2 mg	—

1 ACOG, American College of Obstetricians and Gynecologists; DFE, Dietary Folate Equivalents; NAS, National Academy of Sciences, Engineering, and Medicine; T1, first trimester.

2 Iron specified as elemental iron and used in the following amounts and forms: 300 mg ferrous sulfate hepahydrate, 180 mg ferrous fumarate, or 500 mg ferrous gluconate. Also have an intermittent iron and folic acid supplement recommendation for women who have a hard time ingesting daily and/or in populations were anemia prevalence <20%.

3 For pregnant women who do not ordinarily consume an adequate diet and for those in high-risk categories, such as women carrying >1 fetus, heavy cigarette smokers, and with alcohol and/or drug abuse.

4 ACOG does not provide specific dietary recommendations and appears to get specific nutrient recommendations from RDAs.

Context-specific applications under certain demographic or health conditions are only beginning to be considered. WHO recommendations were recently updated to reflect new evidence relating to micronutrient supplementation including daily iron and folic acid for all pregnant women, calcium in populations with low dietary calcium intake, and vitamin A in areas of endemic vitamin A deficiency, leading to night blindness, where it is a significant public health concern (25, 26).

Outdated guidelines are a further limitation to achieving optimized maternal and fetal nutrition. For example, recent proceedings from an NAS Food and Nutrition Board workshop highlighted the need to update recommendations on nutrition during pregnancy and lactation [released in 1990 (27) and 1991 (28), respectively] due to advancements in reported data and the changing demographics, including the increased prevalence of advanced maternal age and a higher incidence of obesity and type 2 diabetes (T2D) in expectant mothers (15). Moreover, how consumers obtain health information has significantly shifted in the last 2 decades, with social media and the internet now among the leading sources (29). Subsequently, guidelines in their current state may become increasingly limited in their reach. Ultimately, scientific agencies need to be aware of these limitations to ensure that future iterations are applicable and accessible for a broad range of expectant mothers and provide information in an accessible format that is easy to understand.

## Factors Contributing to the Complexity of Achieving Optimal Nutritional Status

Main factors contributing to the challenge of achieving adequate nutritional status during pregnancy and beyond relate to the highly individualized nature of optimum nutrient intake, differences in bioavailability, varying nutritional demands depending on the stage of pregnancy, and the confusing nature of current guidance for dietary supplementation. These challenges are addressed herein.

### Nutrient intake is highly individualized

Nutritional status is governed by numerous interplaying physiological, socioeconomic, cultural, and demographic factors. One of the most fundamental and modifiable determinants of nutritional status is dietary intake, which could vary based on cultural and geographical location. The Global Dietary Database was created in 2010 to assimilate and standardize individual data on dietary factors from different countries and summarized data from studies representing nearly 90% of the global adult population (30). The database highlights how countries face different challenges for achieving adequate intake, which could have significant repercussions for pregnant and lactating women. Pre-existing medical conditions and extremes of maternal weight, such as T2D and obesity, can also have a negative impact on maternal health (31, 32).

Evolving trends in nutrition have led to increased popularity in many different diets, which has added an additional level of complexity to achieving adequate nutrient intake. Globally, veganism, vegetarianism, and gluten-free diets attract the most attention (33); the number of Americans who are vegan has increased from 290,000 in 2004 to more than 9.7 million in 2019 (34). Changes to the food supply have also prompted countrywide adaptations to dietary composition, such as the increase in availability of animal products and sugar in South Korea, China, and Taiwan (35). However, when following specific diets or during periods of altered dietary intake, it is vital that individuals ensure adequate nutrient intake to avoid unwanted adverse effects of malnutrition, especially when certain food groups are omitted (36). Taking the example of veganism and vegetarianism mentioned above, consumers of such diets have an altered risk of imbalances in iron, vitamins B-12 and D, protein, iodine, omega-3 fatty acids, and calcium (37).

Consideration of balanced nutrient intake is more crucial when the physiological demands of pregnancy require additional nutritional support (38). Conversely, consumer trends and/or implementation of dietary changes might precede the formalization into scientific recommendations and could potentially bring improvements in health or pregnancy outcomes. Scientific communities therefore have a responsibility to explore and understand the potential implications of consumer trends and dietary changes, particularly in crucial life stages such as pregnancy.

### Bioavailability exhibits interindividual variability

When providing nutritional guidance during pregnancy, a challenge with the “one-size-fits-all” approach is that nutrient bioavailability—that is, the proportion of nutrients that are absorbed and metabolized—exhibits a high degree of interindividual variability (39). Variability can be attributed to numerous interplaying factors including the individual's base nutritional status, disease state, and genetics (40). Considering these influences when developing generic nutrient recommendations can be challenging, particularly when establishing DRIs and upper limits, as individuals can respond differently to the same nutritional intake and dietary interventions (41, 42). Early results from a study investigating the response to food in a cohort of volunteers from the United States and United Kingdom found that metabolic responses to food were highly individualized and were only partially determined by genetics, with fat and carbohydrate metabolism displaying substantial variation, even between identical twins. The gut microbiome was a key modulator of nutrient bioavailability, which influenced postprandial responses, suggesting a potentially informative strategy for developing personalized nutrition (43). Consistent with other studies, the findings emphasized that nutritional guidance in its current generic state was not sufficient to adequately support subpopulations and individual needs, thereby highlighting the importance of personalizing diet strategies to optimize nutrition. Individual differences in the bioavailability of nutrients from supplements based on consumer characteristics also need to be considered by manufacturers when developing products for use during pregnancy (44).

### Nutrient requirements vary by pregnancy phase

When providing nutritional guidance during the maternal journey, it is important to consider the different phases (preconception, pregnancy, and lactation) to ensure the additional physiological requirements of the mother and offspring are appropriately met at the right time (45, 46).

#### Preconception

Adequate maternal nutrition during the preconception period influences reproductive health and pregnancy outcomes (47) and positively impacts the growth and long-term health of offspring by improving cognitive development and potentially helping to reduce the risk of developing obesity (48–50). Many leading public health bodies, HCPs, and RDNs recommend that women supplement with folic acid for several months during the preconception period, with data suggesting a 50–70% reduction in the risk of neural tube defects in the offspring (51). To ensure this recommendation is received, a dual approach focusing on women and couples most likely to become pregnant and simultaneously promoting health in all women of childbearing potential is the best approach (48). Despite the importance of the preconception period, studies investigating maternal nutrition often initiate during or after the first trimester; thus, there is limited evidence on how this period specifically impacts infant outcomes.

Within the United States, approximately 15% of couples experience infertility (52), with a greater percentage experiencing subfertility. The nutritional status of women prior to conception may play an integral part of conception success. Nutrients that have been described to support fertility include vitamins B-12 and B-6, vitamin D, calcium, ɷ-3 fatty acids, iodine, and selenium (53, 54); however, no clear guidelines on nutrient intake to enhance fertility exist, and future trials are required to define related benefits (53).

#### Pregnancy

Addressing inadequacies in overall health and nutrition prior to pregnancy would be beneficial but is often unrealistic. In high-income countries, as many as 47% of pregnancies are unplanned (55), and women may not prioritize future maternal health in advance of pregnancy. Once pregnant, women are more motivated to implement lifestyle or dietary modifications; thus, pregnancy may act as a prompt to induce positive behavior change (56). A focus on diet in the first trimester may be an appropriate way to eliminate risks associated with poor nutritional status in these women. Although recommendations vary by country, there often exists guidance around optimal gestational weight to support fetal growth, which is dependent upon maternal BMI prior to pregnancy (57). In healthy mothers with a normal prepregnancy BMI, it is generally accepted that energy intake remains the same or similar to prepregnancy requirements during the first trimester. In the United States, no increase in calorie intake is recommended; the European Food Safety Authority (EFSA) recommends an additional 70 calories/d; and international guidelines recommend a small daily increase of 1 g protein (58). During the second trimester, the United States and EFSA recommend an additional 340 or 260 calories/d, respectively, and both increase recommendations in the third trimester to meet the recommended gestational weight gain (additional 452 and 500 calories/d, respectively) (59, 60). Due to the rate of fetal growth, published nutrient recommendations are broadly similar in the second and third trimesters; as an example, additional iron is required to support higher fetal RBC mass and placental development (61). Thus, when counseling women on nutrition during pregnancy, it is important to be attentive to the variation across trimesters and consider prepregnancy BMI when tailoring recommendations.

#### Lactation

For women who breastfeed, it is important that nutrient requirements during lactation are met to ensure adequate milk composition to support early infant development and the additional physiological strain on the mother. Nutrient requirements during this period therefore depend on the ability to breastfeed, the stage of lactation, as well as the volume and composition of milk produced to support infant demand, which averages around 780 mL/d (28, 62). Global nutrient recommendations during lactation vary, and few clear targets have been identified (63). However, with the exception of iron, it is generally accepted that demands for most nutrients and energy are higher than prepregnancy requirements. Iron recommendations are lower during lactation due to the expectation that iron will not be lost due to amenorrhea during the first few months postpartum, and the recycling of iron from stores accumulated during the formation of maternal RBCs will replenish supply (64). An example of nutrient recommendations during lactation compared with prepregnancy and pregnancy requirements, as determined by NAS, is presented in Table 2.

### Current supplementation practice is suboptimal

Prenatal supplements are commonly consumed by expectant mothers to address nutrient imbalances and support optimal maternal and infant outcomes (65). However, recommendations are based on prevention of established and commonly encountered effects of dietary deficiencies and are often unable to close all the gaps in nutritional status for some important nutrients in expectant and lactating mothers. Some countries implement commercial and industrial food-fortification programs to enhance the nutrition obtained from commonly consumed foods like flour and bread. Evidence shows that mandatory folic acid fortification in high-income countries prevents a high percentage of neural tube defects (66, 67), whereas the beneficial impacts of iodine fortification of bread have been identified as being greater in pregnancy and postpartum women than in the general population (68). Fortification is a strategy that could improve nutritional status on a generalized, large scale, particularly in low-income countries, but caution must be taken in pregnant and lactating women to ensure that intakes are not exceeded when supplements may be being used in conjunction.

Although prenatal supplements are commonly recommended and consumed (65), several studies have highlighted nutrient gaps that continue to exist between actual intake and recommendations (11, 45). In a clinical observation, approximately 20–30% of 563 pregnant women were reported to have some form of vitamin deficiency detected by blood tests, despite regular consumption of multivitamin/mineral supplements (45). Similarly, 24-h dietary recall data from the 2001–2014 US NHANES demonstrated that more than 10% of pregnant women were not consuming adequate key nutrients (e.g., iron; folate; vitamins A, D, and E; magnesium; and calcium); moreover, these nutrient deficiencies were found despite approximately 70% of women reporting the use of dietary supplements (11). Nutrient intake adequacy for vitamin K, potassium, and choline did not appear to be influenced by supplementation, while supplementation increased the risk for exceeding the Tolerable Upper Intake Levels for folic acid, iron, and zinc (11). However, it is important to note that traditional methods to measure nutritional status such as 24-h dietary recall, FFQs, and food records are limited due to the subjective nature of data collection (69), reporting bias (70), and insufficient characterization of certain nutrients in food-composition tables (e.g., trace elements such as iodine) (71). These limitations should be acknowledged when interpreting data using such methods.

Women's risk for inadequate nutrient intake during pregnancy can be influenced by demographics, age, and weight status. A recent secondary analysis using 24-h dietary recall and FFQ data of pregnant women from observational cohorts in the US Environmental Influences on Child Health Outcomes Consortium identified subpopulations at particular risk for inadequate intake from foods and supplements (72). Younger women (14–30 y) were less likely to meet calcium, copper, magnesium, phosphorus, vitamin K, and potassium DRIs compared with women older than 30 y (*P* < 0.0001). Non-Hispanic White women were more likely to meet requirements for vitamin E, calcium, copper, magnesium, vitamin K, and potassium compared with other races/ethnicities (*P* < 0.0001). Women who were obese were less likely to meet recommendations for magnesium, vitamin K, and potassium compared with their counterparts who were of normal weight or overweight (*P* < 0.0001). Thus, taking the United States as an example, it appears that existing supplementation strategies to improve maternal nutritional status are often inadequate. Providing a more personalized approach should be considered to best support both the mother and developing fetus.

## Strategies Moving Forward: Personalized Nutrition to Meet Individual Demands

**FIGURE 1:**
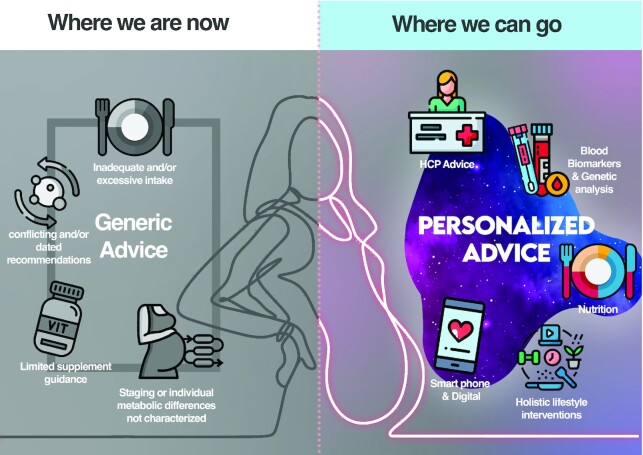
The advancement from current to future nutrition strategies during pregnancy: a focus on personalized advice. Existing strategies to improve nutrition during pregnancy are generic and do not consider the specific demands of either individuals or specific subpopulations. Future strategies could focus on personalized dietary recommendation and supplementation, other holistic lifestyle interventions, digital technologies, blood biomarkers, and genetic analysis. HCP, health care professional.

Benefits of personalized approaches on health-related behavioral changes have been demonstrated outside of pregnancy (73). For example, the “Food4Me” trial in Europe is one of the largest randomized controlled trials to have investigated the efficacy of personalized nutrition in over 1600 participants across 7 countries. Interventions included conventional dietary advice, individualized diets, and diets based on phenotype and genotype. The findings demonstrated the benefits of a tailored approach over a “one-size-fits-all” approach in increasing acceptance and adherence to improved nutritional choices, including reduced consumption of saturated fat and increased consumption of folate (74). Similar strategies could also be used to improve maternal nutrition during pregnancy, with a particular focus on personalized dietary recommendation and supplementation, other holistic lifestyle interventions, digital technologies, blood biomarkers, and genetic analysis (**Figure 1**).

### Towards personalized nutrition

#### Personalized supplementation

Despite limited agency recommendations, reported supplement use in pregnancy is between 47% and 98% in pregnant women across the United States, Australia, and Canada (75–77). However, manufacturers often design products with numerous micronutrients at levels near 100% of the daily recommended value, as the most practicable mass consumer levels. These fail to acknowledge the specific demands of either individuals or specific subpopulations. One and the same broad-spectrum multivitamin/mineral supplement is unlikely to adequately meet the needs of the broad range of all consumers.

**FIGURE 2:**
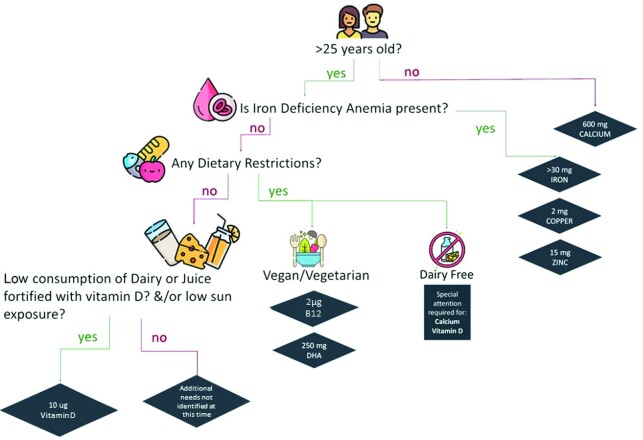
Example “decision tree” to guide supplement choice in pregnant women in the United States. Simple tools like decision trees could be used by pregnant women to guide whether additional supplement support is recommended, based on their current conditions and lifestyle factors. This example was developed based on supplementation recommendations reported by the National Academy of Sciences, Engineering, and Medicine for the US population. B12, vitamin B-12.

By considering the variety of individual factors that influence nutrition, simple tools, frameworks, or decision-making support could enable pregnant women to identify whether supplementation is necessary for them. **Figure 2** shows an example of a “decision tree” that could be used by pregnant women to help guide whether additional support is recommended, based on their current conditions and lifestyle factors. Of note, the presented decision tree was developed based on supplementation recommendations reported by the NAS for the US population (78) but would need further refinement and validation prior to being practically applied. By examining current dietary intakes by subgroups (dietary habits, lifestyle, and chronic health conditions), tools like these could be developed further, both for the US population and on a global scale.

#### Tailored holistic interventions

Optimizing maternal health encompasses far more than the use of supplements to address inadequacies, with an individual's broader diet and lifestyle choices also playing a significant role. Motivation to engage in adequate nutrition has been shown to vary according to factors such as age, life stage, and BMI (48, 79), and is a major barrier to successful implementation of diet and lifestyle interventions. Recent studies have found that personalized strategies have demonstrated improved adherence to healthy diet and lifestyle choices, particularly in women with prominent health concerns such as obesity, T2D, and gestational diabetes. These strategies included individual counseling programs (80), active education programs (81), dietitian-led behavior-change workshops (82), and multicomponent strategies targeting dietary intake, personalized dietary guidance, tailored recipes, and goal sharing (83). The results demonstrated that tailored, holistic approaches have the potential to achieve dietary improvements that are beneficial for both the mother and offspring. Considering a broader picture of maternal health and the associated interconnected factors seems to be more effective when targeting engagement, thereby improving long-term compliance in subgroups of pregnant women who have previously struggled to optimize their health.

### Relevant information to support optimized approaches

#### Digital technologies

In a world where “big data” has become more accessible, digital technologies are emerging that utilize large datasets to both provide better insights and greater accessibility, leading to accumulative health benefits for individuals and populations (84). While studies investigating the effects of application (app)-based and other digital interventions to improve diet and lifestyle during pregnancy are limited, several examples exist (85). In the Netherlands, a randomized controlled trial investigating the effectiveness and compliance rate of a mobile app (Smarter Pregnancy; Erasmus MC, Rotterdam) that provided personalized online coaching to women contemplating pregnancy or in their first trimester of pregnancy improved compliance and nutritional behaviors compared with the control group; a statistically significant reduction in dietary risk score (ranging from 0 = healthy to 9 = unhealthy) was detected in the intervention group at 24 wk (β = 0.75; 95% CI: 0.18, 1.34) compared with a control group (β = 0.550; 95% CI: 0.253, 0.859) (86). Another randomized controlled trial investigated the effects of a digital, guided counseling program in 78 pregnant women in France over a 12-wk intervention period (87). Tailored counseling using a computer-based algorithm was more effective than generic counseling in improving nutrition adequacy midpregnancy and exhibited improvements in the probabilities of adequacy for many essential nutrients including α-linolenic acid (18:3n−3), thiamin, folate, and cholesterol. These results were particularly notable in women who had an initial diet quality score below the population median.

Online platforms have been used for many years to guide dietary recommendations; however, many of these are in the context of weight loss/optimization and lifestyle changes relating to fitness (e.g., MyFitnessPal, Livestrong.com, Weight Watchers). With appropriate implementation, online platforms designed to guide personalized nutrition specifically through pregnancy and lactation could provide a cost-effective, simple, and scalable solution to bring a level of personalized nutrition for expectant mothers with internet access. Pooling large datasets obtained from online and digital platforms could also help establish a clearer picture of what is “normal” for women in different subpopulations; therefore, resources could be created from such datasets to give women advice tailored to their needs (88).

#### Biomarkers

In pregnancy, accurate determination of individual nutritional status is paramount to identify malnutrition or other concerns that may pose a risk to either the mother or infant, and to yield data that forms the basis of broader nutritional guidance. Traditional methods such as 24-h dietary recall, FFQs, and food records (89, 90) have well-documented limitations (91–93) but are often used to collect data that underpin existing and often ineffective nutritional guidance. Nutritional biomarkers are biological parameters that reflect dietary intake and metabolism, acting as a surrogate to provide an objective, quantifiable means of assessing overall nutritional status (94). Therefore, they have a higher degree of sensitivity than traditional methods for nutrient determination (69). For example, urinary nitrogen is one of the most commonly used biomarkers, and 24-h measurements can be collected to validate dietary intake of protein (95). Although the potential role of nutritional biomarkers in determining prenatal health and improving pregnancy outcomes is not yet well established and often limited to specific nutrients, foods, and dietary patterns, future research looks promising. One particularly interesting method could be the use of metabolomics in nutrition epidemiology to decipher the interactions between diet, health, and disease more clearly (96). Although more research is required, robust blood biomarkers could be used in clinical research to understand the implications of dietary patterns in subpopulations of women during pregnancy more accurately.

#### Genetic analysis

Common genetic variation in the form of single nucleotide polymorphisms (SNPs) can influence the expression of genes responsible for metabolism, leading to dietary intolerances and nutrient or vitamin inefficiencies. Termed “nutrigenetics,” this specific area of genetic research is a tool that could be successfully harnessed to provide individualized nutrition advice (97, 98). Common genetic variants have been associated with reduced nutrients that are key for fertility and fetal health, such as vitamins B-12 (99) and D (100), folate (101), choline (102), and iron (103). At present, evidence is lacking for specific gene-related nutrient recommendations in routine clinical practice. However, carriers of risk variants may benefit from additional emphasis on the importance of meeting the RDA in pregnancy to ensure adequate nutrient recommendations are met.

A nutrient that has been researched with respect to genetic status and maternal health is DHA, a long-chain PUFA important for neurological development. DHA can be obtained by consuming oily fish, for example, or by endogenous synthesis from ɑ-linolenic acid (an essential fatty acid) in a process that is regulated by fatty acid desaturase (FADS) enzymes. Associations between maternal or infant status of *FADS* genes and cognitive outcomes of offspring have been demonstrated (104, 105). Furthermore, genetic variants in *FADS1* and *FADS2* have been associated with reduced blood and breast-milk fatty acid concentrations in pregnant and lactating women (106–108). Mothers genotyped as homozygous for the rs174575-G SNP have been shown to have significantly reduced DHA concentrations in breast milk compared with rs174575-C homozygotes (106). Furthermore, an observational study reported that consumption of up to 3 portions of fatty fish per week raised plasma DHA but failed to increase milk DHA in rs174575-G homozygotes compared with CC mothers (107). This finding was recently replicated in 191 Taiwanese mothers stratified by DHA intake and an *FADS* risk score (combining 2 SNPs in the *FADS* locus, rs1535 and rs174448) (108). These studies may suggest impaired or reduced translocation of DHA into breast milk for women with *FADS* risk variants. Investigation into whether a higher dose of DHA supplementation (e.g., 1000 mg/d) can overcome this genetic predisposition is warranted. Many diet–gene interactions have been described for a number of nutrition phenotypes. With further research, integration of blood biomarker and genetic analysis techniques into routine practice could be instrumental in the prevention and treatment of noncommunicable diseases from the prenatal development stage and beyond (109).

### Real-world practical application beyond diet

One of the main barriers to successful implementation of diet and lifestyle interventions lies with participant engagement and adherence. Recent studies have found that personalized strategies improved adherence to healthy diet and lifestyle choices, particularly in women with prominent health concerns such as obesity, T2D, and gestational diabetes. These strategies included individual counseling programs (80), active education programs (81), dietitian-led behavior-change workshops (82), and multicomponent strategies targeting dietary intake, personalized dietary guidance, tailored recipes, and goal sharing (83). The results demonstrated that tailored, holistic lifestyle interventions have the potential to achieve dietary improvements that are beneficial for both the mother and offspring. These approaches may be increasingly feasible considering the constant emergence of new data and technological advances, offering a real opportunity for future strategies, recommendations, and health information to become more individualized and yield better results. Considering a broader picture of maternal health and the associated interconnected factors seems to be more effective when targeting engagement, thereby improving long-term compliance in subgroups of pregnant women who have previously struggled to optimize their health.

## Discussion and Future Directions

A lack of understanding and consensus of what constitutes adequate maternal nutrition makes optimizing maternal nutrition difficult. One of the primary limitations to public health education and guidance is the population-based “one-size-fits-all” approach. This type of approach has provided women with generic advice that often fails to consider the vast array of individual factors that affect individual nutritional status; consequently, there is substantial variation in the health of expectant mothers and their offspring (110).

Personalized nutrition offers women the opportunity to optimize their health by using strategies that are appropriate and tailored to their unique nutritional needs. However, there are several challenges and limitations associated with moving towards personalized nutrition. One of the primary concerns lies with the cost and complexity of implementing personalized strategies into routine practice. While a global interest, working toward adequate nutritional status in pregnant women is difficult as much of the data underpinning our current understanding have been obtained from high-income countries, or fail to acknowledge the interplaying physiological, socioeconomic, cultural, and demographic factors that prevent women from accessing the information and support they need. Extensive and costly research will be required to establish the benefits of personalized nutrition strategies in the first place. Once established, some strategies may be relatively low cost to implement (e.g., guidance on the risks of excess nutrient intake), whereas others will be high cost (e.g., the use of blood biomarkers and genetic analysis to guide dietary choices). This could be a hurdle, particularly in low-income countries or in those that lack the infrastructure to enable sufficient delivery. Digital and app-based technologies, for example, will not be of widespread use in regions where internet access is not routinely available, whereas strategies that harness this technology could prove a cost-effective solution with extensive mobile phone and computer ownership. Although online and app-based technologies are offering quick, simple, and far-reaching access to information, many freely available resources are unregulated and limited research has been conducted to assess their quality, which could give consumers false or misleading information (111). The potential for poor regulation also applies to blood biomarker and genetic analysis tests, in which consumers may be led towards unnecessary investigations or subjected to tests with poor analytical performance. Existing and future developments in these fields should only be recommended after close collaboration with nutrition experts to ensure that the available information is scientifically robust and validated. Another challenge lies with the relatively short time frame of pregnancy, and whether personalized nutrition strategies are sufficient to yield substantial improvements to maternal and/or fetal health across this period. The investment of resources required to deliver personalized strategies may be high; for example, some strategies could involve a higher level of HCP/RDN support. Global and regional agencies may therefore conclude that a more general approach to nutrition can be sufficient, and that a more personalized approach would not justify the resources required to achieve it.

The future of more personalized approaches for optimized maternal nutrition relies on several factors. First, robust research must be conducted to establish nutritional requirements across the whole spectrum of pregnant women worldwide, which, in turn, will yield accurate datasets to develop personalized nutrition strategies. Many nutrition studies do not collect information on demographic and socioeconomic factors (e.g., ethnicity, income), although these are strongly associated with disparities in adherence to nutritional guidelines and maternal health (112). This hinders the application of data to inform strategies for minority populations or women who have unique nutritional needs. Second, studies should compare the cost-effectiveness, practicality, and efficacy of these strategies versus existing methods (e.g., individual supplementation vs. large-scale food-fortification programs). Third, nutritional experts must collaborate with relevant governing and commercial agencies to facilitate appropriate dissemination of research data, establish improved guidelines, develop personalized strategies, and tailor education for women appropriately on these specified guidelines. Women living in poverty, for example, have a higher risk of poor nutrition and may need access to food-assistance programs or consumer education programs (113), whereas women with a higher income are more likely to take dietary supplements but may require further guidance on the risks of over-supplementation (114). Fourth, there is a need for greater transparency and regulation of dietary supplements. Not all countries have robust legislation in place, which could result in misleading marketing and improper labeling and leave consumers vulnerable to scams. Historically, in the United States, there have been concerns over the lack of premarket registration of dietary supplements, as well as notable inconsistencies in the response to adulterated, counterfeit, and imported products (115). Finally, expert agencies should aim to improve adherence to nutritional advice. Deviating from expert advice could be due to a variety of reasons, including difficulty in interpreting nutritional guidelines, lack of relevance to personal circumstances, and limited broader knowledge surrounding maternal health and nutrition (116). Overall, an integrated cross-sector approach is vital to equip relevant agencies with the information required to translate into validated, robust, and truly representative guidelines and nutritional strategies that are not only more accessible but also applicable and thereby more effective for a broader range of pregnant women and their individual circumstances.

## Conclusions

Existing evidence supports the idea that tailored approaches for improving maternal nutrition are important and could potentially improve maternal and offspring health on a much broader scale than is currently attainable; however, these approaches must be viable for their intended population. A greater understanding of differences in nutrition at both the individual and subpopulation level is warranted to ensure that pregnant women receive information that is appropriate for their needs (117). Future efforts should aim to obtain data that can evaluate the wider implications of tailored strategies—including cost-effectiveness, ease of implementation, and the effect on pregnancy outcomes—while also assessing the feasibility of integrating such interventions into current guidance and practice. Proactively reviewing emerging data, and orchestrating interactions between governing agencies, regulators, consumer organizations, and nutritional experts including food industry representatives, could form a multifaceted approach to developing practical solutions to tackle inadequate nutrition, thereby ensuring the best possible support for all women during preconception, pregnancy, lactation, and beyond.

## Notes

The publication fees and medical writing support for this manuscript were funded by Reckitt/Mead Johnson Nutrition.

Author disclosures: The authors are employees of Reckitt, a consumer health company.

Abbreviations used: ACOG, American College of Obstetricians and Gynecologists; app, application; EFSA, European Food and Safety Authority; FADS, fatty acid desaturase; HCP, health care professional; NAS, National Academy of Sciences, Engineering, and Medicine; RDN, Registered Dietitian Nutritionist; SNP, single nucleotide polymorphism; T2D, type 2 diabetes.
